# Nutraceutical Molecules Slow Down Retinal Degeneration, in Tvrm4 Mice a Model of Retinitis Pigmentosa, by Genetic Modulation of Anti-oxidant Pathway

**DOI:** 10.3389/fnins.2022.868750

**Published:** 2022-04-19

**Authors:** Ilaria Piano, Francesca Corsi, Beatrice Polini, Claudia Gargini

**Affiliations:** ^1^Department of Pharmacy, University of Pisa, Pisa, Italy; ^2^Department of Surgical, Medical and Molecular Pathology and Critical Care Medicine, University of Pisa, Pisa, Italy; ^3^Interdepartmental Center for Nutraceutical Research and Nutrition or Health, University of Pisa, Pisa, Italy

**Keywords:** nutraceutical treatment, photoreceptors degeneration, genetic modulation, oxidative stress, retinitis pigmentosa

## Abstract

Rhodopsin (RHO) mutations are responsible for 25–40% of the dominant cases of retinitis pigmentosa (RP) with different severity and progression rates. The Tvrm4 mice, heterozygous for an I307N dominant mutation of RHO, display a normal retinal phenotype when raised in ambient light conditions, but undergo photoreceptor degeneration when briefly exposed to strong white light. Here, The Tvrm4 mice is pre-treated with naringenin 100 mg/kg/die, quercetin 100 mg/kg/die, naringenin 50 + quercercetin 100 mg/kg/die or vehicle dimethyl sulfoxide (DMSO 0.025%) in the drinking water for 35 days. On the 30th day, retinal degeneration was induced by exposure for 1 min to the white light of 12,000 lux intensity, and the treatment was repeated for another 5 days. At the end of the protocol retinal functionality was tested by recording an electroretinogram (ERG). The retinal tissue was collected and was used for further analyses, including immunohistochemically, biochemical, and molecular biology assays. The data obtained show that treatment with nutraceutical molecules is effective in counteracting retinal degeneration by preserving the functionality of photoreceptors and increasing the antioxidant and anti-apoptotic pathways of retinal cells. The present data confirm that nutraceutical molecules are effective in slowing photoreceptor degeneration in a mutation-independent way by modulating the antioxidant response of the retina at the gene expression level.

## Introduction

In recent years, the role of oxidative stress in the progression of neurodegenerative diseases, also due to specific genetic mutations, has become increasingly important (Bacci et al., 2021). The oxidative stress is due to the disruption of a delicate balance between free radicals of oxygen or nitrogen and the presence of antioxidant molecules and involves the accumulation of radical species that are intrinsically unstable thus leading to the oxidation of molecules in the cellular environment (Castelli et al., 2021). The small amounts of reactive oxygen species (ROS), generated by nicotinamide adenine dinucleotide phosphate (NADPH) oxidases within the cytoplasm, or by oxygen–electron mismatch donors present in the mitochondria, are neutralized by glutathione and by enzymes of the superoxide dismutase (Sod) family. However, under pathological conditions, such as high oxygen levels or other sources of free radical generators, the antioxidant system may be insufficient to cleanse the environment from ROS, leading to the production of radicals even more harmful to the cell, such as hydroxyl radicals. When the free radicals encounter macromolecules, they produce the characteristic modifications that compromise lipids, proteins, and DNA constituting oxidative damage (Campochiaro and Mir, 2018). In the mouse retina, rods make up 97.2% of cells in the outer nuclear layer and present a high number of mitochondria that make them highly metabolically active (Jeon et al., 1998).

In diseases characterized by primary rod death as in retinitis pigmentosa (RP), there is a progressive reduction in oxygen consumption as a result of rod death, leading to an increase in oxidative stress that, in a second phase of the disease, contributes to the death of cones. The hypothesis that oxidative stress is the initial cause of cone degeneration, independently of the type of genetic mutation that underlies the primary degeneration and death of the rods, is supported by studies showing that the use of antioxidant molecules is effective in slowing the death of cones in several models of RP, including rd1, Q344ter, and rd10 mice (Komeima et al., 2007, 2008; Oveson et al., 2011; Piano et al., 2019) and P23H rats (Fernández-Sánchez et al., 2012).

Here, we demonstrate how two nutraceutical molecules, naringenin a bioflavonoid compound present in high concentrations in the citrus species (Viswanatha et al., 2017) and quercetin a flavonoid present in various vegetables; tea and red wine (D’Andrea, 2015; Ortega and Jastrzebska, 2021), administered individually or in combination, can modulate genes encoding for key proteins involved in the antioxidant response in an animal model of autosomal dominant RP, Tvrm4 (Budzynski et al., 2010; Gargini et al., 2017). This study adds to those already in the literature demonstrating that natural molecules can slow retinal degeneration in genetic models of RP (Liu et al., 2021) and induced models (Kim et al., 2015).

## Materials and Methods

### Animals

Animals were treated according to the guidelines of the Declaration of Helsinki and according to Italian and European institutional guidelines, following experimental protocols approved by the Animal Welfare Organization (OPBA – Ethics Committee) of the University of Pisa and the Italian Ministry of Health (Protocol #653/2017-PR-DB173.3.EXT.0, Department of Pharmacy, University of Pisa). Heterozygous Tvrm4 (RhoTvrm4/Rho+) mice on a C57Bl/6J background, were selected by genotyping (Budzynski et al., 2010) and males and females in the same ratio were used. All animals were regularly fed *ad libitum* and housed in a controlled lighting environment (12-h light/dark cycle), with an illumination level of less than 60 lux. All experiments were conducted under deep anesthesia by intraperitoneal (i.p.) injection of 20% urethane in saline buffer (0.9% NaCl) at a dose of 0.1 ml/10 g body weight. At the end of the experimental procedure, the mice were sacrificed by cervical dislocation while maintaining the deep anesthesia condition, and the tissues were collected for *ex vivo* analysis.

**FIGURE 1:**
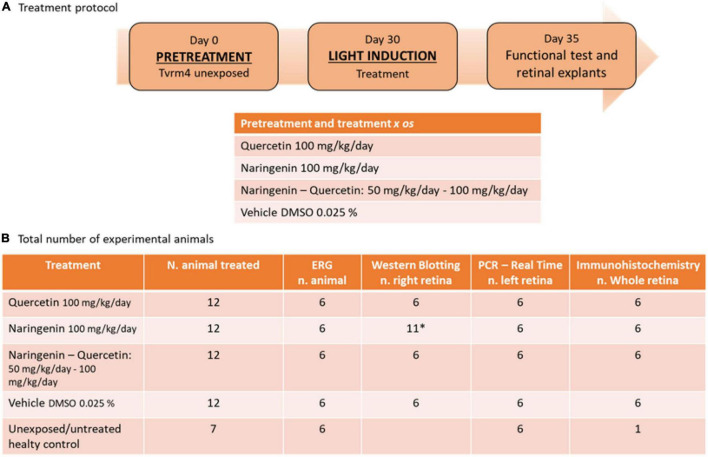
Treatment protocol and the number of animals used for the experimental plane. **(A)** Summary outline of the treatment protocol and the relative doses used for treatment with single molecules and treatment with molecules in combination. **(B)** Descriptive table of the total number of animals used for this study, with a detailed breakdown of how biological samples were used. *Due to the variability of the results of this treatment *n* = 5 contralateral retinas were taken from the animals designated for whole-mount retinal tissue preparation (right eye).

The treatment protocol used and the total number of animals divided by the experimental techniques are shown in Figure 1.

### Treatment

The experimental protocols started when the animals were approximately 4–5 months old, in agreement with previous studies performed in the same mouse model (Piano et al., 2020). The individually housed animals were treated for 35 days with solutions of naringenin 100 mg/kg/day, quercetin 100 mg/kg/day, naringenin 50 + quercetin 100 mg/kg/day or vehicle (DMSO 0.025%).

The chosen dose of the single-administered molecules is derived from experimental evidence showing a reduced biological effect at lower doses (data not shown) and good efficacy at the doses proposed in previous studies conducted in the same laboratory (Piano et al., 2019, 2021) and by other colleagues (Testai et al., 2013). To evaluate a potential interaction between the two phenolic compounds, the ability of the less active (naringenin) to potentiate the effects of the most effective molecule (quercetin) has been analyzed. Therefore, quercetin is administered at the dose corresponding to that of the single treatment, while naringenin is administered at an ineffective dose (data not shown). The treatment was divided into two phases. During the first phase, Tvrm4 unexposed were pre-treated for 30 days; on day 30, they were exposed to light according to the protocol (Gargini et al., 2017); the second treatment phase lasted 5 additional days. The stock solutions of naringenin (45 mg/ml) and quercetin (10 mg/ml) were prepared in DMSO and added to the volume of the drinking water according to the bodyweight of the animal to achieve the expected daily dose.

### Electroretinogram

The general procedure for animal preparation, anesthesia, electroretinogram (ERG) recording, light stimulation, and data analysis has been described in detail previously (Piano et al., 2021). Briefly, ERGs were recorded in complete darkness using coiled gold electrodes contacting the cornea moisturized by a thin layer of gel (Lacrinom, Farmigea), and the reference (earth) electrode was inserted at scalp level. The animal was placed inside the Ganzfeld sphere 30 cm in diameter, whose interior surface was coated with a highly reflective white paint and exposed to light stimulation. The light stimulation was carried out with a white light electric flash (SUNPACK B3600 DX, Tecad Company, Tokyo, Japan) and, to modulate the intensity, six calibrated neutral density filters were used. For scotopic ERG recordings, the mice were presented with a single flash of increasing intensity (1.71 × 10^–5^ to 377 cd*s/m^2^, 0.6 log units steps), each repeated six times, with an inter-stimulus interval ranging from 20 s for dim flashes to 45 s for the brightest flashes. Isolated cone (photopic) components were obtained by superimposing the test flashes (0.016 to 377 cd*s/m^2^), on a steady background of saturating intensity for rods (30 cd/m^2^) after at least 15 min from background onset.

The amplitude of the scotopic a-wave was measured at 7 ms after the onset of the light stimulus and the b-wave was measured from the peak of the a-wave to the peak of the b-wave. The amplitude of the photopic b-wave was measured from the baseline to the peak of the b-wave. The data were analyzed with the LabVIEW 2019 program (National Instruments, Austin, TX, United States).

### The mRNA Expression Analysis

The purification and extraction of total RNA from retina tissues were performed by miRNeasy Micro Kit (Qiagen, Hilden, Germany) according to the manufacturer’s instructions. The extracted RNA was quantified by the Infinite M200 NanoQuant instrument (Tecan, Salzburg, Austria) and retro-transcribed by RT2 First Strand Kit (Qiagen, Hilden, Germany). The obtained cDNA was used for real-time polymerase chain reaction (PCR) expression analysis of multiple genes by using RT2 Profiler PCR Array (Mouse Oxidative Stress and Antioxidant Defense, #330231, PAMM-065Z, Qiagen, Hilden, Germany). The expression analysis was carried out by the Gene Globe Data Analysis Center (^1^ Qiagen, Hilden, Germany). For the normalization step, the software automatically selected the most stable genes among those analyzed as the optimal set of reference genes. The geometric mean of these stable genes was used as a normalization factor.

In the same cDNA samples, the transcriptional levels of genes coding photoreceptors were further analyzed by real-time PCR by using specific primers RT^2^ qPCR Primer Assay (Catalog No. 330001, Qiagen, Hilden, Germany) and RT2 SYBR^®^ Green qPCR Mastermix (Qiagen, Hilden, Germany).

The selected primers were as follows: Guanine nucleotide-binding protein alpha transducing 1 (GNAT1): GeneGlobe ID – PPM03513A-200, Detected Transcript NM_008140; arrestin 3, retinal (ARR3): GeneGlobe ID – PPM04815A-200, Detected Transcripts NM_133205; guanine nucleotide-binding protein, alpha transducing 2 (GNAT2): GeneGlobe ID – PPM06964A-200, Detected Transcripts NM_008141; cyclic nucleotide-gated channel beta 1 (CNGB1): GeneGlobe ID – PPM06962A-200, Detected Transcripts NM_001195413. The obtained data were normalized over the same normalization factor used to analyze data from array experiments.

The normalized expression value is expressed as fold-change relative to the control group (DMSO-treated animals).

### Western Blotting

After the ERG recordings, retinas were extracted and lysed with the modified RIPA buffer, described by Piano et al. (2013), and proteins were quantified with the Bradford assay. A 20-μg of total protein, mixed with 2× Laemmli, was loaded for each sample into pre-cast 4–20% polyacrylamide gels (Mini-PROTEAN TGX gel, Bio–Rad). After the electrophoretic run, the gel was activated using the ChemiDoc™ XRS + instrument (Bio–Rad, California), and the separated proteins were transferred onto polyvinylidene fluoride (PVDF) membranes (Trans–Blot Turbo PVDF Transfer packs, Bio–Rad). The proteins were acquired to normalize the intensity of the investigated band with total protein (Gürtler et al., 2013). The blocking step was performed with EveryBlot Blocking Buffer solution (Biorad), 5 ml × 5 min at room temperature, to prevent non-specific antibody binding. The membrane was then incubated with the primary antibody (Table 1) diluted in 5 ml of the same solution, for 1 h at room temperature. After 5 × 5 min washes in t-TBS, the membrane was incubated with the secondary antibody conjugated to the peroxidase enzyme (HRP), in the same manner as the primary; washed (5 × 5 min) with t-TBS and then the immunoblot signal was visualized by using an enhanced chemiluminescence substrate detection system (LuminataTM Forte Western HRP Substrate, Millipore). The chemiluminescent images were acquired by Chemidoc XRS+ (Bio–Rad). The quantification was performed with Image Lab Software 6.0 (Bio–Rad). The proteins band intensities were normalized to amount of total protein in corresponding lane utilizing stain-free technology (Gürtler et al., 2013).

**TABLE 1:** List of antibodies for Western blotting and immunohistochemistry.

Antibody	Host	Company	Work dilution	Application
Anti-RHO	Mouse monoclonal	Sigma-Aldrich	1:1000	WB
Anti-opsin Red/Green	Rabbit polyclonal	Merck Millipore	1:500	WB
Anti-opsin Blue	Rabbit polyclonal	Merck Millipore	1:500	WB
Anti-Sod1	Rabbit polyclonal	Merck Millipore	1:1000	WB
Anti-Sod2	Goat polyclonal	Merck Millipore	1:1000	WB
Anti-Sod3	Mouse monoclonal	Merck Millipore	1:1000	WB
Anti-Caspase3	Rabbit polyclonal	Merck Millipore	1:1000	WB
Anti-Sirt1	Mouse monoclonal	Merck Millipore	1:1000	WB
Anti-cone arrestin	Rabbit polyclonal	Merck Millipore	1:500	IH
Anti-rabbit IgG HRP conjugated		Merck Millipore	1:5000	WB
Anti-mouse IgG HRP conjugated		Sigma-Aldrich	1:5000	WB
Anti-goat IgG HRP conjugated		Sigma-Aldrich	1:5000	WB
Anti-rabbit (Alexa Fluor 568)		Merck Millipore	1:500	IH

### Immunohistochemistry

After the enucleation, the eye was marked dorsally, the anterior segment removed, to obtain the eyecup and fixed in 4% paraformaldehyde in phosphate saline buffer (PBS) for 20 min at room temperature, followed by 3 × 10 min washes in PBS. Subsequently, the retina was separated from the pigmented epithelium and placed in “free-floating.” After that, the blocking step was performed in 5% bovine serum albumin (BSA) in PBS + Triton 0.1% at 4°C. The next day, the retina was incubated with the primary antibody (Table 1) in 1% BSA + Triton 0.1% for 3 days at 4°C. At the end of the 3 days, after 3 × 10 min washes in PBS, the retina was incubated with the secondary antibody for 2 more days. Finally, after an additional 3 × 10 min washes in PBS, the retina was mounted on the slide with the photoreceptor side up and covered with VECTASHIELD and coverslip. The images were acquired with a fluorescence microscope Nikon model, NiE with digital camera Nikon model, DS-Ri2. The several images acquired were joined through the use of Nis software to recreate the entire retinal surface and with the same software was also traced and measured the damaged area.

### Statistical Analysis

The statistical comparisons for ERG registrations and WB results were carried out with one-way analysis of variance (ANOVA test), followed by *t*-tests with Bonferroni correlation, using the Origin Lab 8.0 program (MicroCal, Northampton, MA, United States). For mRNA expression analysis, the statistical comparisons were performed using Student’s *t*-test of the normalized values for each gene in treatment groups compared to DMSO treated group. The *p*-values less than 0.05 were considered statistically significant.

## Results

**FIGURE 2:**
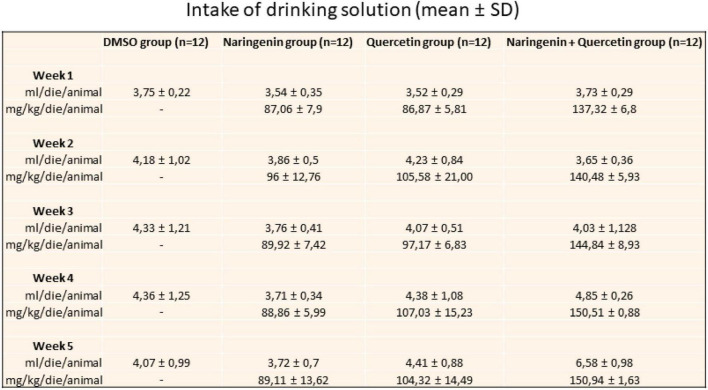
Daily intake of drinking solution. The table shows the value expressed in mean ± SD of the relative intake for a week for each group of treatment.

The effectiveness of the sub-chronic treatment with naringenin or quercetin (100 mg/kg/day) and naringenin 50 + quercetin 100 mg/kg/day on the Tvrm4 induced mice (1 min 12,000 lux) retinal function was assessed by a variety of functional, morphological, and biochemical/molecular biological tests. Figure 2 shows the daily intake of the solution and the effective dose of molecules taken for each group of mice treated.

First, the retinal function was assessed by scotopic and photopic ERG to evaluate the viability of rods and cones, respectively, in the three groups treated with nutraceutical molecules and compare with their respective controls Tvrm4 induced who received DMSO *per os* only. The tissues were then analyzed using immunohistological techniques for investigations regarding morphology and real-time PCR and western blot techniques for the determination of expression of photoreceptors and markers of oxidative stress to evaluate the presence of proteins essential for the survival of photoreceptors, oxidative stress, and apoptosis.

### Functional and Morphological Recovery of Retinal Neurons After Administration of Natural Molecules

**FIGURE 3:**
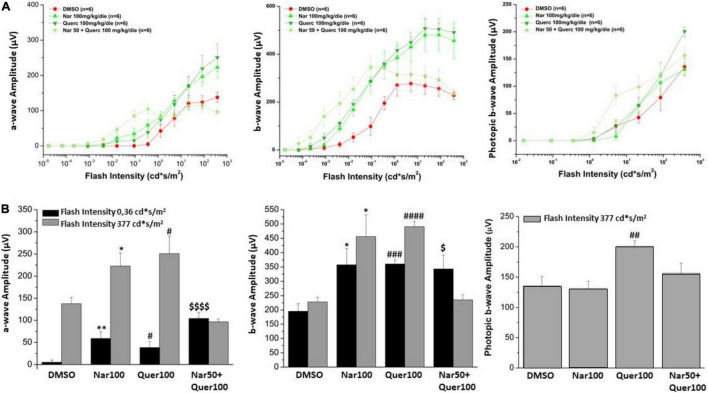
Retinal function was assessed by scotopic and photopic ERG recording. **(A)** Scotopic a- and b-wave amplitude and photopic b-wave amplitude as a function of light stimulus intensity, in treatment (green scale line) and control groups (red line). **(B)** Histogram for the amplitude of scotopic a- and b-waves at a low light intensity (0.36 cd*s/m^2^, black bars) and the highest (377 cd*s/m^2^, gray bars); the amplitude of photopic b-wave at the highest light intensity (377 cd*s/m^2^, gray bars). Bars represent mean ± SEM; statistical analysis was performed using one-way ANOVA test followed by Bonferroni test (*p ≤ 0.05, ^**^p ≤ 0.01 naringenin 100 group vs. DMSO group; ^#^*p* ≤ 0.05, ^##^*p* ≤ 0.01, ^###^*p* ≤ 0.001, ^####^*p* ≤ 0.0001 quercetin 100 group vs. DMSO group; ^$^*p* ≤ 0.05, ^$$$^*p* ≤ 0.001, naringenin 50 + quercetin 100 group vs. DMSO group).

The alteration in retinal function and morphology, between the unexposed/untreated healthy control group and mice exposed to the light induction that triggers the genetic mutation that causes retinal degeneration, is shown in Supplementary Figure 1; Figure 3A shows the light intensity/response curves of the ERG after the sub-chronic treatments *per os* with naringenin 100 mg/kg/day, quercetin 100 mg/kg/day, naringenin 50 + quercetin 100 mg/kg/day, and of the control group, respectively, of the scotopic and photopic ERG. Figure 3B shows the histograms of the scotopic and photopic ERG’s waves amplitude at the lowest luminance (0.36 cd*s/m^2^, black bars) and the most intense light stimulus (377 cd*s/m^2^, gray bars). From the graphs, it is possible to note, concerning the scotopic ERG, that in the case of the single treatment (naringenin 100 mg/kg/day or quercetin 100 mg/kg/day) the amplitude is significantly higher than in the group of animals treated with the vehicle alone. The combined treatment (naringenin 50 + quercetin 100 mg/kg/day) does not show the same trend. In this case, the scotopic ERG shows a greater response of the rods to less intense light stimuli (as if the light sensitivity were increased) compared to the control, this response; however, seems to reach saturation at lower light intensity also compared to the other experimental groups analyzed. In the photopic ERG, the effect obtained does not show statistically significant differences between the treatment and the control groups, except for the single treatment with quercetin 100 mg/kg/day.

**FIGURE 4:**
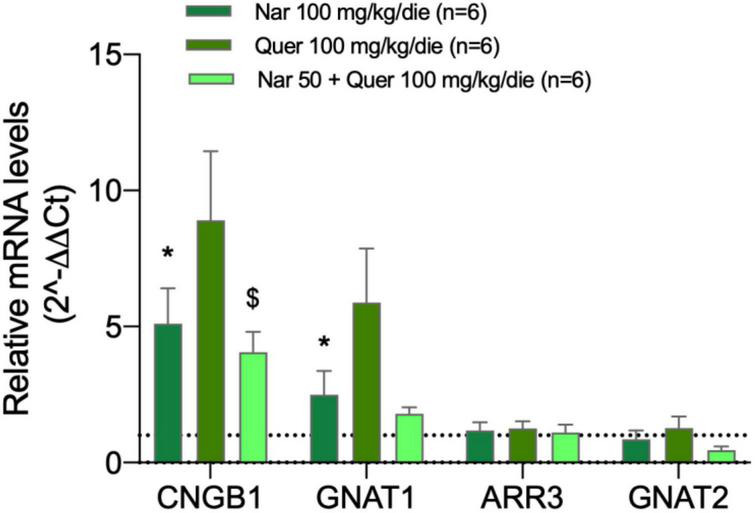
Transcriptional levels of photoreceptor genes from the treated groups of animals compared to the pathological control. The transcriptional levels of four mRNAs coding for photoreceptor proteins were analyzed by real-time PCR. Specifically, the histogram shows the expression of CNBG1 coding light-sensitive channel, GNAT1 coding transducin 1, ARR3 coding cone-arrestin, and GNAT2 coding transducin 2 in the control group (DMSO treated animals) and in groups treated with naringenin 100 mg/kg/day, quercetin 100 mg/kg/day, and naringenin 50 + quercetin 100 mg/kg/day (*n* = 6, respectively). The data are representative of six animals ± SEM. The bars represent the value obtained after normalization of each value on the mean expression of the most stable genes among those analyzed (from array data) and are expressed as fold-change relative to the control group (made = 1). The statistical analysis was performed using Student’s *t*-test of the normalized values for each gene in treatment groups (*p ≤ 0.05, naringenin 100 group vs. DMSO group; ^$^*p* ≤ 0.05, naringenin 50 + quercetin 100 group vs. DMSO group).

Figure 4 shows the bar graph of gene expression specific for photoreceptors of mice treated with nutraceutical molecules, all samples of treated mice are normalized to the control (dashed line). The analyzed genes encode for proteins responsible for the proper functioning of photoreceptors: CNBG1 (light-sensitive channel), GNAT1 (transducin 1) present in the rods, and ARR3 (cone–arrestin), GNAT2 (transducin 2) present in the cones. The graph shows the ability of compounds to strongly modulate the transcriptional expression of genes present at the level of rods in mice: naringenin and quercetin induce an increase in the expression of CNGB1 and GNAT1.

**FIGURE 5:**
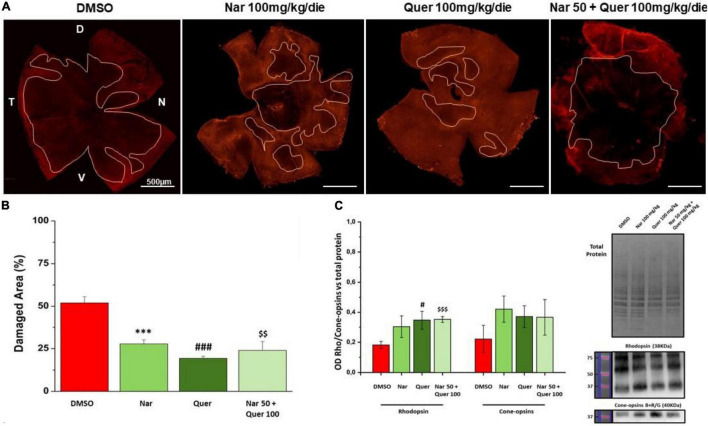
Morphological assessment of the damaged area. **(A)** Morphology of whole-mount retina of naringenin 100 mg/kg/die, quercetin 100 mg/kg/die, naringenin 50 + quercetin 100 mg/kg/die treated groups and pathological control (cone-arrestin, red). The pink line delineates the damaged area after photo-induction. **(B)** Histogram for quantification of the damaged area as a percentage of total retinal area for each treatment and control. Each treatment group used to study retinal morphology consisted of *n* = 6 animals. The bars represent mean ± SEM; the statistical analysis was performed using one-way ANOVA test followed by Bonferroni test (^***^p ≤ 0.001, naringenin 100 group vs. DMSO group; ^###^*p* ≤ 0.001, quercetin 100 group vs. DMSO group; ^$$^*p* ≤ 0.01, naringenin 50 + quercetin 100 group vs. DMSO group). **(C)** WB analysis for the treatment and control groups. On the right, the image of the bads related to total protein, RHO, and cone–opsins blue + red/green; on the left, the histogram related to the quantification, by optical densitometry, of RHO and cone–opsins. The number of animals used for WB analysis was, respectively, DMSO (*n* = 6); naringenin 100 mg/kg/die (*n* = 11); quercetin100 mg/kg/die (*n* = 6); naringenin 50 + quercetin 10 mg/kg/die (*n* = 6). The data are representative of animals ± SEM. The bars represent each value normalized with the total protein and then compared with the control group (DMSO only); the statistical analysis was performed using one-way ANOVA test followed by Bonferroni test (^#^*p* ≤ 0.05, quercetin 100 group vs. DMSO group; ^$$$^*p* ≤ 0.001, naringenin 50 + quercetin 100 group vs. DMSO group).

Figure 5 shows the results obtained through morphological analysis of the retina and the assessment of protein levels (RHO and cone–opsins) essential for rods and cones. In panel A, are illustrated reconstructions of retinas labeled for cone–arrestin (red) and the pink line delineates the damaged areas (black areas) after photo-induction in treated animals compared to the group that received vehicle alone. In Figure 5B, the bar graph shows that the reduction in the percentage of damaged retinal area in treated mice is statistically significant compared with the control mice (*naringenin 100 vs. DMSO; # quercetin 100 vs. DMSO; $ naringenin 50 + quercetin 100 vs. DMSO). Figure 5C shows the results obtained by WB for the quantification of the protein levels of RHO (38 kDa) and cone–opsins blue + red/green (40 kDa). From the bar graph, it is possible to notice that RHO protein levels are higher in treated mice compared to the control (red bar) with significant results for the single treatment quercetin (100 mg/kg/day) and the combined treatment (naringenin 50 + quercetin 100 mg/kg/day) (#quercetin 100 vs. DMSO; $ naringenin 50 + quercetin 100 vs. DMSO). On the other hand, the results obtained from the quantification of cone–opsins blue + red/green were not significant.

### Efficacy of Natural Molecules in Modulating Oxidative Stress Defense and Apoptosis in Retinal Tissue

**FIGURE 6:**
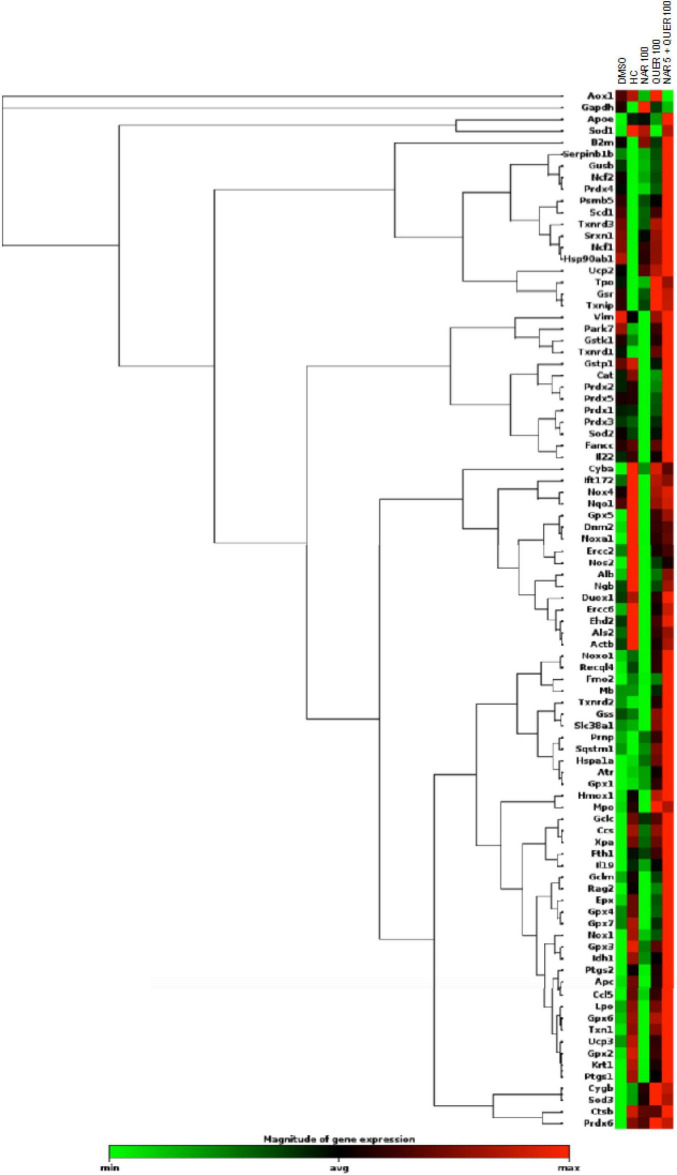
Heatmap of genes (mRNAs) involved in oxidative stress and antioxidant response. Columns reports the transcriptional expression levels of 84 genes in groups treated with DMSO alone (control group), naringenin 100 mg/kg/day, quercetin 100 mg/kg/day, and naringenin 50 + quercetin 100 mg/kg/day (*n* = 6, respectively). Furthermore, the expression levels of the same genes in HC (*n* = 6). Each square represents the mean values of the values obtained after normalization on the mean expression of the most stable genes among those analyzed. Red color indicates the most upregulated expression levels, while green color indicates the least downregulated expression levels.

After verifying the viability and survival of photoreceptors following treatments with antioxidant nutraceuticals, the effect on markers of oxidative stress and apoptosis was evaluated. In RP, following the death of rods, triggered by the genetic mutation present, is created at the level of retinal tissue a condition of hyperoxia that causes an increase in the production of ROS (Yu et al., 2000, 2004). The presence of ROS and the consequent increase in oxidative stress is considered one of the factors leading to degeneration and secondary death of cones (Valter et al., 1998; Cingolani et al., 2006; Komeima et al., 2006, 2007, 2008; Lee et al., 2011). The previous studies in the rd10 animal model (autosomal recessive RP) showed a reduction in Sod1 and Sod2 following treatment with naturally occurring antioxidants (naringenin and quercetin) (Piano et al., 2019). Through the use of real-time PCR and WB technique, gene expression and protein levels of enzymes involved in cellular pathways aimed at counteracting oxidative stress were determined. As shown in Figure 6, the analysis of 84 genes, shows that the treatment with antioxidant molecules induces changes in the regulation of several genes involved in oxidative stress and antioxidant response. The gene regulation profile after naringenin and quercetin treatments shows a different color pattern in the heatmap (Figure 6), where quercetin seems to lead to greater changes than naringenin in the expression of genes involved in oxidative stress and antioxidant response. In detail, naringenin leads to upregulation of 10 genes (fold-change ≥ 2 vs. DMSO, used as pathological control; data not shown) and downregulation of 4 genes (fold-change ≤ 0.5 vs. DMSO; data not shown). The treatment with quercetin affected the expression of 32 genes, whose transcriptional levels appear strongly increased (fold-change ≥ 2 vs. DMSO; data not shown). Interestingly, the combined treatment appears able to affect the expression of almost all analyzed genes: 45 genes show a great increase in their expression levels (fold-change ≥ 2 vs. DMSO, data not shown), exhibiting an opposite profile of gene expression compared to DMSO treated group (Figure 6).

**FIGURE 7:**
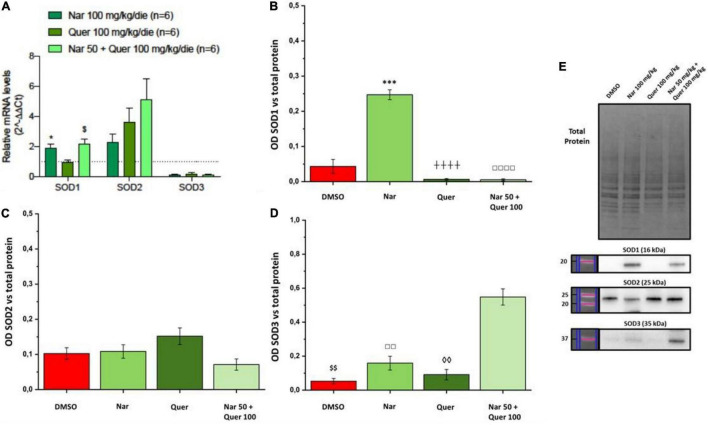
Real-time PCR and WB analysis of oxidative stress markers. **(A)** Trend and statistical significance of transcriptional expression of Sod1, Sod2, and Sod3 genes (data from array analysis) in groups treated with naringenin 100 mg/kg/day, quercetin 100 mg/kg/day, and naringenin 50 + quercetin 100 mg/kg/day (*n* = 6, respectively) compared to the control group (*n* = 6). The data are extrapolated from the array analysis and are representative of six animals ± SEM. Bars represent the value obtained after normalization of each value on the mean expression of the most stable genes among those analyzed (from array analysis). The data are expressed as fold-change relative to the control group (made = 1). The statistical analysis was performed using Student’s *t*-test of the normalized values for each gene in treatment groups (*p ≤ 0.05, naringenin 100 group vs. DMSO group; ^$^*p* ≤ 0.05, naringenin 50 + quercetin 100 group vs. DMSO group). **(B–D)** Quantification by WB of the protein levels of Sod1, Sod2, Sod3 in the groups treated with naringenin 100 mg/kg/day (*n* = 10), quercetin 100 mg/kg/day (*n* = 6) and naringenin 50 + quercetin 100 mg/kg/day (*n* = 4) compared with the control group (DMSO, *n* = 6). **(E)** Image of the bands related to Total Protein, Sod-1, Sod-2, and Sod-3. The data are representative of animals ± SEM. The bars represent each value normalized with the total protein and then compared with the control group (DMSO only); statistical analysis was performed using one-way ANOVA test followed by Bonferroni test (^***^p ≤ 0.001, naringenin 100 group vs. DMSO group; ^$$^*p* ≤ 0.01, naringenin 50 + quercetin 100 group vs. DMSO group; ^++++^*p* ≤ 0,0001, naringenin 100 group vs. quercetin 100 group; ^◊◊^*p* ≤ 0.01, naringenin 50 + quercetin 100 group vs. quercetin 100 group; ^□□^*p* ≤ 0.01, ^□□□□^*p* ≤ 0.0001, naringenin 100 group vs. naringenin 50 + quercetin 100 group).

**FIGURE 8:**
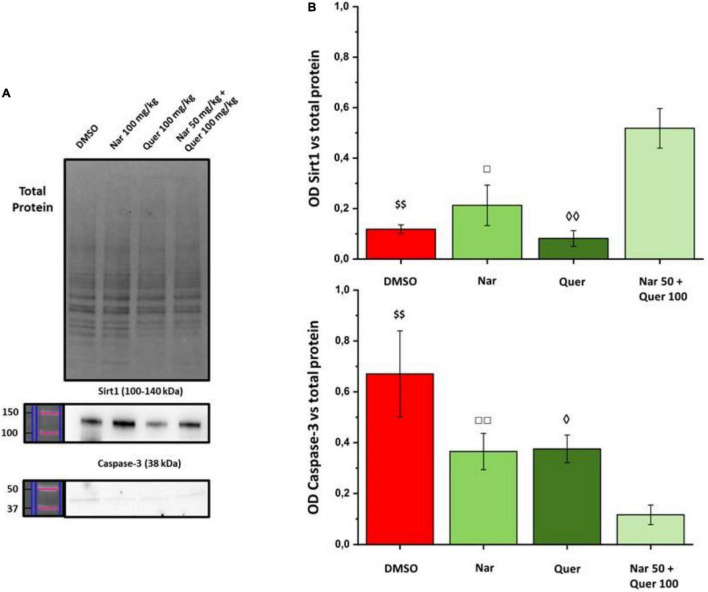
WB analysis of apoptosis markers. **(A)** Image of the bands related to Total Protein, Caspase-3, and Sirt-1. **(B)** Histogram relative to the quantification, by optical densitometry, of Caspase-3 and Sirt-1. The number of animals used for WB analysis were, respectively: control group (DMSO, *n* = 6); naringenin 100 mg/kg/day (*n* = 11); quercetin 100 mg/kg/day (*n* = 6); naringenin 50 + quercetin 100 mg/kg/day (*n* = 6). The data are representative of animals ± SEM. The bars represent each value normalized with the total protein and then compared with the control group (DMSO only); the statistical analysis was performed using one-way ANOVA test followed by Bonferroni test (^$$^*p* ≤ 0.01, naringenin 50 + quercetin 100 group vs. DMSO group; ^◊^*p* ≤ 0.05, ^◊◊^*p* ≤ 0.01, naringenin 50 + quercetin 100 group vs. quercetin 100 group; ^□^*p* ≤ 0.05, ^□□^*p* ≤ 0.01, naringenin 100 group vs. naringenin 50 + quercetin 100 group).

To better understand these modulations, the transcriptional levels of the same genes were analyzed also in healthy control (HC) mice. Compared to pathological control, the expression of 43 genes is strongly modulated (fold-change ≥ 2 or fold-change ≤ 0.5 vs. DMSO) in HC, suggesting a role of these genes in the pathogenesis of RP. As shown in Figure 5, naringenin modulates the expression of some genes oppositely compared to HC. For example, dual oxidase 1 (Duox1), nitric oxide synthase 2 (Nos2) and Uncoupling Protein 3 (Ucp3) have an opposite trend. Instead, both quercetin and the combined treatment show an expression profile closer to that of HC compared to naringenin (Figure 6). For example, mice treated with quercetin and the combination show a similar expression profile of gene family coding glutathione peroxidases, lactoperoxidase, myeloperoxidase, and NADPH oxidases and their activators, compared to HC. Noteworthy, after the combined treatment, the transcriptional levels of several genes are strongly over-expressed compared to HC: the expression of genes coding glutathione peroxidases (Gpx), interleukin 19, recombination activating gene 2 (Rag2), and RecQ protein-like 4 (Recql4) is doubled. Furthermore, many genes (Gpx), lactoperoxidases (Lpo), prostaglandin–endoperoxide synthase 2 (Ptgs2), or myeloperoxidase (Mpo) are increased not only compared to HC but also to quercetin. Overall, quercetin appears the treatment closest to HC. Figure 7A shows the fold-change of the transcriptional expression of SOD1, SOD2, and SOD3 after treatment with antioxidants, normalized to the control group. Figures 7B–D show the protein levels of Sod1, Sod2, Sod3 enzymes, respectively, in treated mice compared with the control group treated with vehicle (red) Figure 7E shows a representative example of a WB experiment. The transcriptional expression of the SOD1 gene (A) is significantly upregulated after naringenin and the combined treatment (*naringenin 100 vs. DMSO; $naringenin 50 + quercetin 100 vs. DMSO;1.88 ± 0.64 and 2.18 ± 0.73-fold-increase, respectively). The SOD2 gene expression is increased after all treatments, as opposed to SOD3 expression (A). The results show reduced protein levels for Sod1 (B) in the combined and single treatment with quercetin compared to controls, in contrast, the single treatment with naringenin shows a significant increase in protein levels compared to the control (*naringenin 100 vs. DMSO; + naringenin 100 vs. quercetin 100; □ naringenin 50 + quercetin 100 vs. naringenin 100). The histogram for Sod2 (C) shows no statistically significant differences in the levels of the enzyme in the retina of treated mice compared to controls. The bar graph in D relative to Sod3, data showing, in all the various types of treatment, an increasing trend in the levels of the enzyme in treated animals compared to the control (red). Sod3 levels increased significantly ($DMSO vs. naringenin 50 + quercetin 100; □ naringenin 50 + Quercetin 100 vs. naringenin 100; ◊ naringenin 50 + Quercetin 100 vs. quercetin 100) in the group of animals that received the combined treatment (naringenin50 + quercetin 100 mg/kg/day) compared to the control group. To assess whether the administered nutraceutical molecules also could interfere with photoreceptor death, protein levels of markers of the apoptotic pathway were assessed (Figures 8A,B). In particular, Sirt-1, an anti-apoptotic factor, and Caspase-3, the ultimate effector of the cascade of apoptosis and common to the extrinsic and intrinsic pathways, were evaluated; the vehicle-treated control group (red bar), as shown by the histograms in Figure 8B, had high levels of the pro-apoptotic factor Caspase-3 and a low amount of the anti-apoptotic factor Sirt-1. From the histograms related to the treatments (green scales) it is evident a significant increase ($ DMSO vs. naringenin 50 + quercetin 100; □ naringenin 50 + quercetin 100 vs. naringenin 100; ◊ naringenin 50 + quercetin 100 vs. quercetin 100) of the anti-apoptotic factor Sirt-1 in the presence of the combined treatment (naringenin 50 + quercetin100 mg/kg/day) compared to the control while no significant variation is present following the single treatment with naringenin (100 mg/kg/day) or quercetin (100 mg/kg/day). Concerning the combined treatment, in accordance with the histogram relating to Sirt-1, there is a significant decrease ($DMSO vs. naringenin 50 + quercetin 100; □ naringenin 50 + quercetin 100 vs. naringenin 100; ◊ naringenin 50 + quercetin 100 vs. quercetin 100) of Caspase-3 compared to the control group (red); even in the single treatment, although not statistically significant, there is still a decrease of Caspase-3 compared to the diseased control.

## Discussion

The recent work in patients suggests that oxidative stress may promote blood–retinal barrier damage by modulating the production of vasoactive factors, and also accelerate the morphological damage of cone photoreceptors. Several studies in the literature have shown that too high concentrations of ROS lead to cellular damage in both animals and humans, in fact in their eyes, high levels of proteins resulting from oxidative damage were detected in the aqueous humor, compared with controls and how also the formation of cataracts in the eyes can be linked to oxidative stress (Campochiaro et al., 2015; Vingolo et al., 2022).

A previous study conducted in our laboratory has shown the predominant role of oxidative stress in secondary cone-photoreceptor death and the role in slowing down the degeneration of antioxidant molecules in an autosomal recessive model of RP (Piano et al., 2019). Further studies, conducted in different animal models of RP, confirmed the strong involvement of oxidative stress in cone degeneration by demonstrating how different therapeutic approaches, which target oxidative stress, are effective in slowing down the progressive degeneration of these cells (Assimopoulou et al., 2005; Shen et al., 2005; Komeima et al., 2006, 2007, 2008; Kanakis et al., 2007; Fernández-Sánchez et al., 2012; Xiong et al., 2015; Wang et al., 2016; Trouillet et al., 2018).

In the present study, we tested a therapeutic strategy using two flavonoids abundant in fruits and vegetables, naringenin and quercetin, to counteract the oxidative stress underlying RP progression. Indeed, these flavonoids show antioxidant properties that interact with the cellular detoxification enzyme system (Al-Dosari et al., 2017). Furthermore, it has been shown that the anti-oxidant and anti-apoptotic effects of flavonoids can limit neurodegeneration by providing neurotrophic support to prevent retinal damage in RP as in other types of retinal disorders, such as diabetic retinopathy (Al-Dosari et al., 2017) and age-related macular degeneration (AMD) (Maccarone et al., 2008; Falsini et al., 2010; Gopinath et al., 2018). Our findings confirm, and extend, previous data published by other groups on the pivotal role of increased oxidative stress in photoreceptor death and the beneficial effects of antioxidant molecules in slowing retinal degeneration. Specifically, we observed that sub-chronic non-invasive treatment with naturally derived molecules, frequently present in normal diets, can significantly slow disease progression in an animal model of autosomal dominant RP, Tvrm4. The single treatment with the two molecules is effective in preserving the functionality and morphology of the retina. Noteworthy, quercetin appears more effective to protect retinal function compared to naringenin. This less neuroprotection could be partly explained by the changes in transcriptional expression of genes involved in antioxidant defense regulation, where naringenin shows the expression profile is more different from HC. Furthermore, both gene and protein expression analysis showed an increase in SOD1 levels, as opposite to quercetin. Even if SOD1 has an important role in the antioxidant defense system in the retina (Dong et al., 2006), overexpression of SOD1 also showed increased oxidative damage and more rapid loss of cone function (Usui et al., 2011). Evidence reports that there is a discrepancy between the activity of the individual natural compound and the activity of whole fruit or vegetable from which they are extracted (Miller and Rice-Evans, 1997; Koss-Mikołajczyk et al., 2019; Baranowska et al., 2020). One of the possible explanations could be a pharmacological interaction between natural compounds. Therefore, we analyzed the potential effects of combined administration of the two flavonoids, evaluating the ability of the less active compound to potentiate the protective effects of the most active molecule. Un-expectedly, the combined treatment of the two molecules showed a different trend from single molecules. The expression analysis of genes involved in oxidative stress and antioxidant response suggests one possible explanation also to the unforeseen trend of the combined treatment: instead of restoring the physiological balance minimizing the levels of ROS, the co-treatment could lead to an excessive antioxidant response reducing also low levels of ROS that are involved in cell signaling and redox regulation, thus inducing an “anti-oxidative stress” (Poljsak and Milisav, 2012; Poljsak et al., 2013; Kornienko et al., 2019). Indeed, both anti-oxidative and oxidative stresses lead to the anti-oxidative imbalance that can be damaging (Poljsak et al., 2013). Moreover, the combined treatment induces a strong increase in the protein expression of SOD3 levels, as opposed to single treatments with naringenin and quercetin. Even if SOD3 protects from oxidative injury, its excessive increase can be deleterious (Ikelle et al., 2021).

Furthermore, from the results obtained it is possible to observe that the response to low luminance of the scotopic ERG, for animals treated with the combination of the two molecules, is greater than in pathological controls. This trend shows a drastic reduction at high light intensities, indicating a phenomenon of saturation of the photoreceptors and the lack of ability to recycle the photopigment (Lamb and Pugh, 2004; Kiser et al., 2012). This could be due to the ability of two molecules in combination to preserve cells from death (by modulation of the anti-apoptotic agent Sirt-1) and to stabilize the RHO molecules still available (Herrera-Hernández et al., 2017; Ortega et al., 2019) but inducing the antioxidant stress. As a result of this state within photoreceptors and RPE cells, which are responsible for recycling retinoid from RHO and are involved in phagocytosis of the tips of the outer segments of photoreceptors, there is an accumulation of toxic factors that do not allow the proper functioning of the photoreceptors. The development of anti-oxidative stress could cause metabolic deficits at the level of photoreceptors that hypothetically are no longer able to recycle and renew the proteins necessary for the proper functioning of the phototransduction machine (Ortega and Jastrzebska, 2019).

## Conclusion

Overall, the data obtained from this study show that the anti-oxidant and anti-apoptotic effects of flavonoids may limit neurodegeneration by providing neurotrophic support to prevent retinal damage in an animal model of autosomal dominant RP (Tvrm4 mice) as in other types of retinal diseases, such as diabetic retinopathy (Al-Dosari et al., 2017) and AMD (Maccarone et al., 2008; Falsini et al., 2010; Gopinath et al., 2018). In this work, we first used the Tvrm4 mouse model of RP to test the potential beneficial effects of two nutraceutical compounds, naringenin, and quercetin in single or combined administration, found in a typical Western diet, using a sub-chronic non-invasive treatment. In conclusion, our results demonstrate that supplementing the diet with sufficient doses of flavonoids could be an effective preventive, mutation-independent, non-invasive approach to slow retinal degeneration. This neuroprotective approach requires further investigation to estimate the true efficacy of the treatment to preserve vision in patients with various forms of RP and to establish the optimized regimen of exogenous antioxidant molecules especially when these are administered in combination.

## Data Availability Statement

The datasets presented in this study can be found in online repositories. The names of the repository/repositories and accession number(s) can be found in the article/Supplementary Material.

## Ethics Statement

The study was reviewed and approved by the Animal Welfare Organization (OPBA - Ethics Committee) of the University of Pisa and the Italian Ministry of Health (Protocol #653/2017-PR-DB173.3.EXT.0, Department of Pharmacy, University of Pisa). This study was conducted according to the guidelines of the Declaration of Helsinki and according to Italian and European institutional guidelines.

## Author Contributions

IP and CG conceptualized the study, contribute to the supervision, and wrote the original draft. IP, FC, and BP contributed to the data curation and formal analysis. CG and IP contributed to the funding acquisition. FC and BP contributed to the methodology. IP, FC, BP, and CG contributed to writing, reviewing, and editing the manuscript. All authors contributed to the article and approved the submitted version.

## Conflict of Interest

The authors declare that the research was conducted in the absence of any commercial or financial relationships that could be construed as a potential conflict of interest.

## Publisher’s Note

All claims expressed in this article are solely those of the authors and do not necessarily represent those of their affiliated organizations, or those of the publisher, the editors and the reviewers. Any product that may be evaluated in this article, or claim that may be made by its manufacturer, is not guaranteed or endorsed by the publisher.

## Footnotes

1
https://geneglobe.qiagen.com

## Supplementary Material

Supplementary Figure 1 Retinal function and morphology. **(A)** Scotopic a- and b-wave amplitude and photopic b-wave amplitude as a function of light stimulus intensity, in Unexposed/untreated health control (black scale line) and Exposed groups (red line). **(B)** Representative ERG traces of scotopic and photopic ERG at the highest light intensity (377 cd*s/m^2^ gray bars); **(C)** Whole-mount retina stained against cone–arrestin protein.

Supplementary Figure 2 Specificity control of cone-arrestin antibody on retinal sections of unstressed/untreated Tvrm4 mice.

Supplementary Figure 3 Control of the specificity of RHO **(A)** and cone–opsin **(B)** antibodies, respectively, on retinal protein lysates and on cell lysates of ARPE-19, a retinal pigment epithelium cell line that physiologically does not express photoreceptor-specific photopigments.
