# Supplementary figures and images for: Nutraceutical Molecules Slow Down Retinal Degeneration, in Tvrm4 Mice a Model of Retinitis Pigmentosa, by Genetic Modulation of Anti-oxidant Pathway
Source: Front Neurosci. 2022 Apr 19;16:868750. doi: 10.3389/fnins.2022.868750 (PMC9063314; doi:10.3389/fnins.2022.868750)

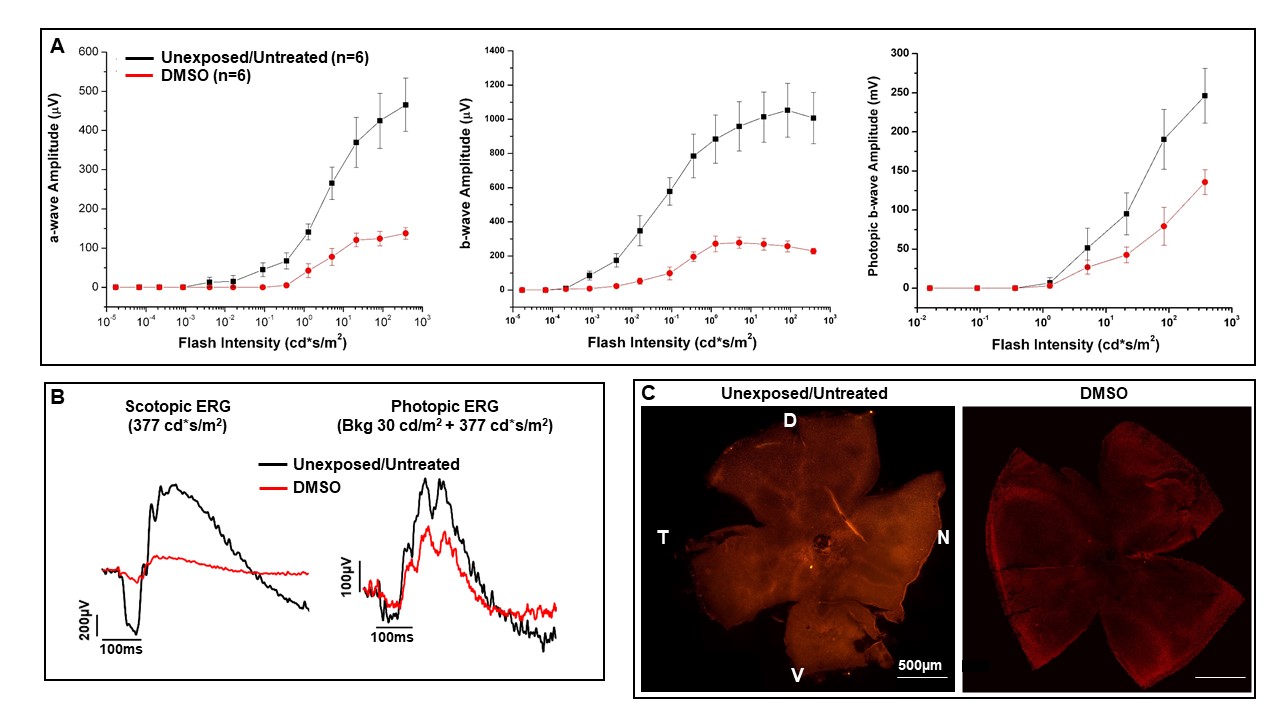

Supplement: Supplementary Figure 1 — Retinal function and morphology. (A) Scotopic a- and b-wave amplitude and photopic b-wave amplitude as a function of light stimulus intensity, in Unexposed/untreated health control (black scale line) and Exposed groups (red line). (B) Representative ERG traces of scotopic and photopic ERG at the highest light intensity (377 cd*s/m2 gray bars); (C) Whole-mount retina stained against cone–arrestin protein. [file Image_1.JPEG]

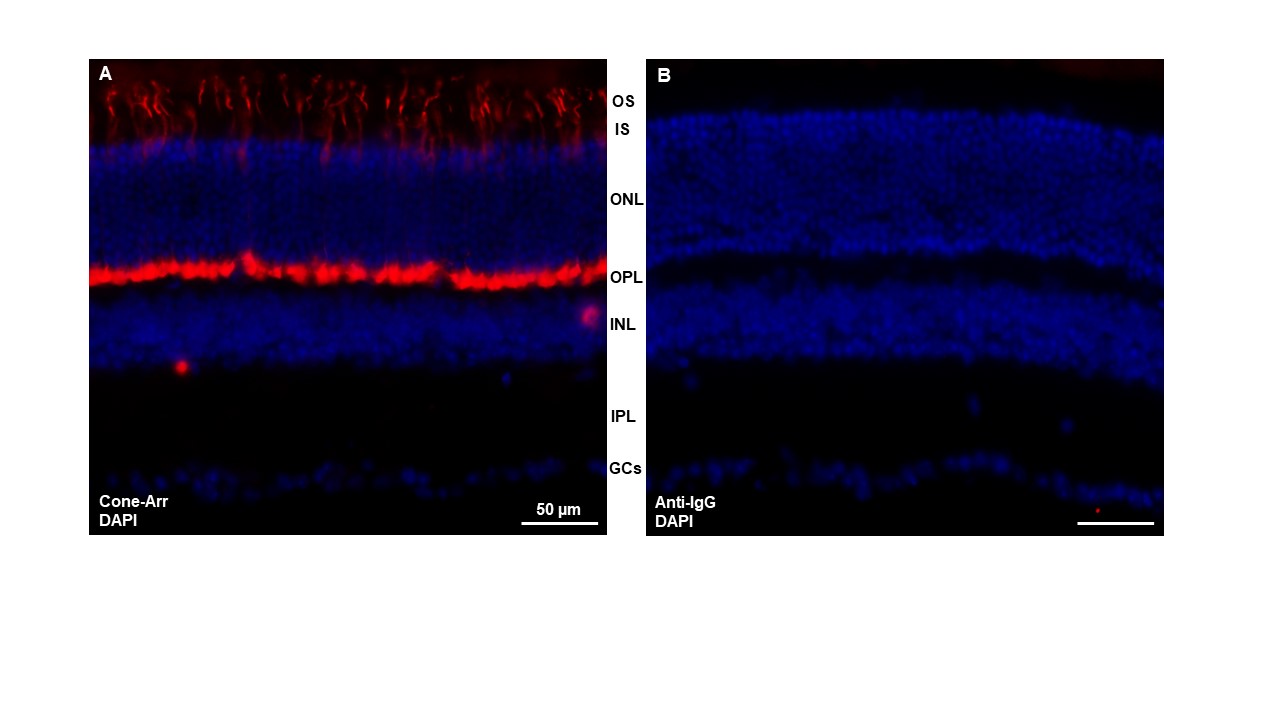

Supplement: Supplementary Figure 2 — Specificity control of cone-arrestin antibody on retinal sections of unstressed/untreated Tvrm4 mice. [file Image_2.JPEG]

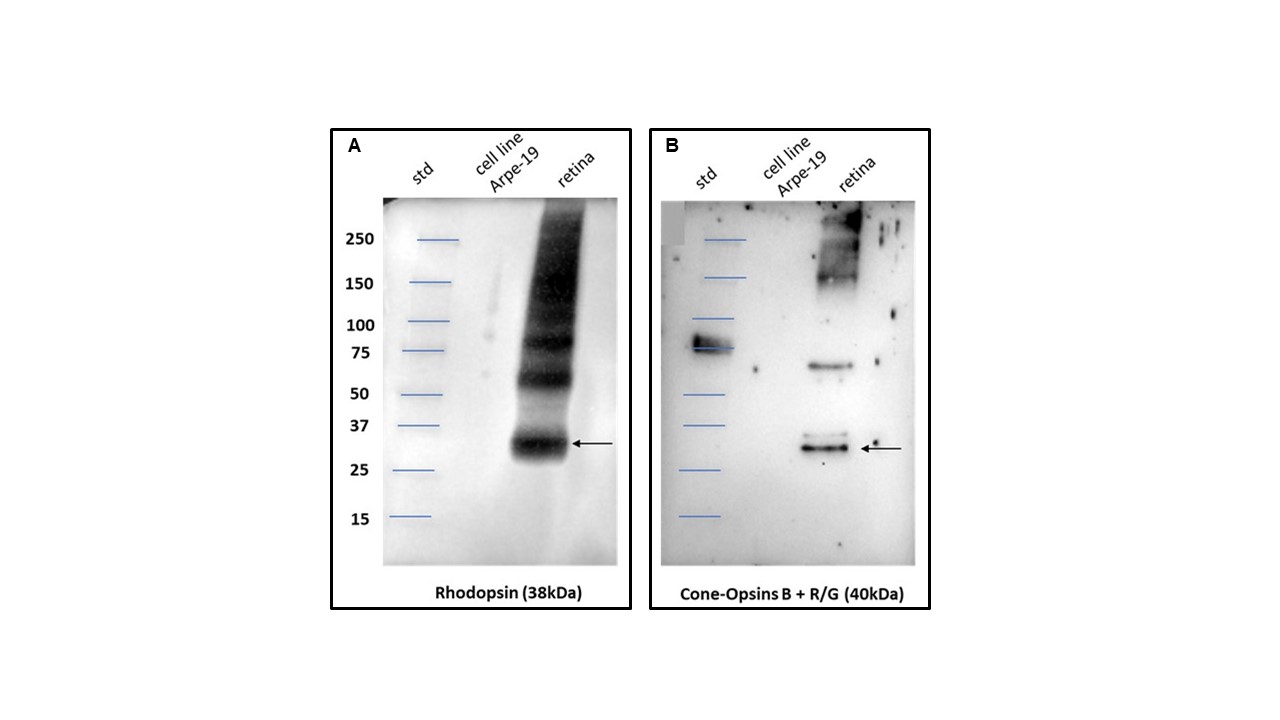

Supplement: Supplementary Figure 3 — Control of the specificity of RHO (A) and cone–opsin (B) antibodies, respectively, on retinal protein lysates and on cell lysates of ARPE-19, a retinal pigment epithelium cell line that physiologically does not express photoreceptor-specific photopigments. [file Image_3.JPEG]
